# Pyridine Based Chalcones: Synthesis and Evaluation of Antioxidant Activity of 1-Phenyl-3-(pyridin-2-yl)prop-2-en-1-one Derivatives

**DOI:** 10.17795/jjnpp-10024

**Published:** 2013-07-17

**Authors:** Mahdi Mojarrab, Roozbeh Soltani, Alireza Aliabadi

**Affiliations:** 1 Department of Pharmacognosy and Pharmaceutical Biotechnology, Faculty of Pharmacy, Kermanshah University of Medical Sciences, Kermanshah, IR Iran; 2 Students Research Committee, Kermanshah University of Medical Sciences, Kermanshah, IR Iran; 3 Department of Medicinal Chemistry, Faculty of Pharmacy, Kermanshah University of Medical Sciences, Kermanshah, IR Iran

**Keywords:** Pyridines, Chalcone, Antioxidants, 6-hydroxy-2,5,7,8-tetramethylchroman-2-carboxylic Acid

## Abstract

**Background:**

Natural chalcones and also their synthetic derivatives have attracted increasing attention due to various pharmacological applications. Development and discovery of new chalcones with antioxidant activities is one of the attracting areas in medicinal and natural product chemistry.

**Objectives:**

In the present study, a new series of pyridine based chalcones was synthesized and their antioxidant capacity was evaluated by beta carotene bleaching (BCB), DPPH free radical scavenging, ferrous ion chelating (FIC) activity and Trolox equivalent antioxidant capacity (TEAC) methods.

**Materials and Methods:**

All compounds were synthesized via an aldol condensation procedure in methanol or ethanol solvent at room temperature and characterization was carried out by ^1^HNMR, IR and MS spectroscopic methods. Related melting points were also measured for each compound.

**Results:**

Fortunately, compounds 3e (16.53 ± 1.21 µg/mL), 3g (58.85 ± 1.10 µg/mL) and 3i (58.73 ± 12.94 µg/mL) showed higher antioxidant activity (EC50 ± SD) in comparison with quercetin (87.24 ± 3.93 µg/mL) as reference agent in ferrous ion chelating method. Furthermore, compounds 3g (4.82 ± 0.11 µg/mL) and 3h (6.33 ± 0.30 µg/mL) also exhibited an acceptable antioxidant property compared to Trolox (3.83 ± 0.22 µg/mL) in TEAC method. None of synthesized compounds demonstrated significant antioxidant activity in DPPH free radical scavenging as well as beta carotene bleaching tests.

**Conclusions:**

According to the obtained data, synthesized pyridine based chalcones (3a-3j) could be proposed as potential antioxidant lead compounds.

## 1. Background

**Figure 1.**
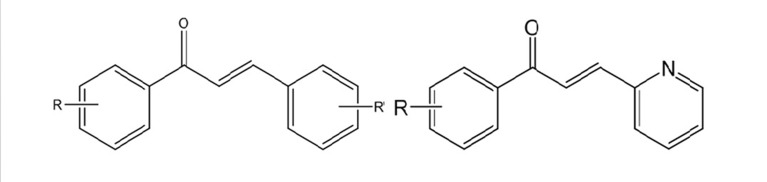
Total Structure of Chalcones

**Figure 2.**
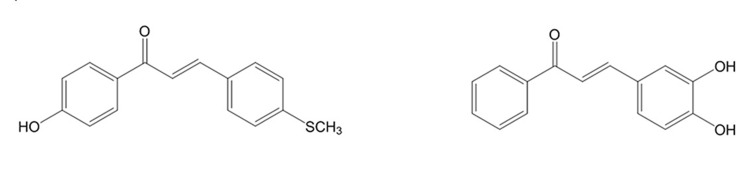
Structures of Two Chalcones With Antioxidant Activity

**Figure 3.**
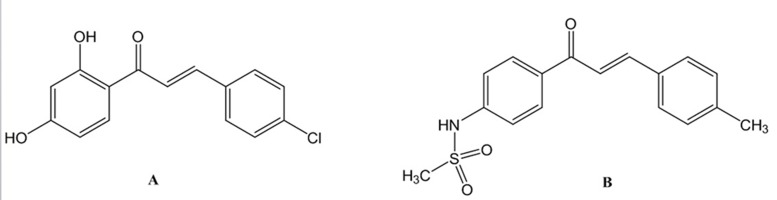
Structures of Some Chalcones as Enzyme Inhibitors A. Monoamine oxidase (MAO) inhibitor, B. Selective cyclooxyganse II (COX-2) inhibitor

**Figure 4.**
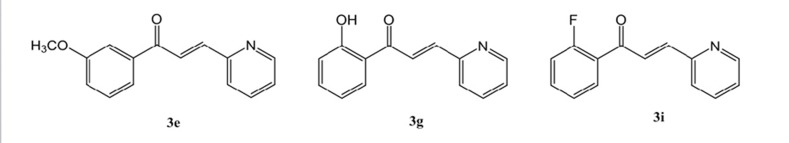
Compounds 3e, 3g and 3i as Potential Antioxidant Agents in FIC and TEAC Methods

Oxygen is an essential factor to many living organisms for the production of energy to fuel biological processes. However, Lferrozine solution (5 mM oxygen leads to the generation of free radicals which induce oxidative damage to some cell compartments like DNA, proteins, membrane lipids and carbohydrates. In recent years, the role of free radicals in the etiology and occurrence of numerous diseases has been disclosed. Neoplastic diseases, diabetes mellitus, cardiovascular diseases, ageing and inflammatory diseases (rheumatoid arthritis) could be mentioned as examples of disorders that are closely related to the free radicals. Antioxidant drugs are capable of protecting cells from the damage induced by free radicals. The antioxidants scavenging the free radicals that generate by biological systems (1). The importance of reactive oxygen species (ROS) has attracted attention globally over the past decade. The human body produces reactive oxygen species such as superoxide anion radical, hydroxyl radical and hydrogen peroxide by many enzymatic systems through oxygen consumption. Larger amounts of these ROS are dangerous because of their ability to attack numerous molecules such as proteins and lipids thereby contributing to more than one hundred disorders in humans (2). It has been proved that oxidative stress has a key role in etiology and occurrence of a wide range of diseases consisting Alzheimer's disease, multiple sclerosis (MS), cancer, immunologic disorders, rheumatologic diseases, cardiovascular disorders (atherosclerosis) and cerebrovascular accident (CVA). Oxidative stress could lead to the DNA damages (both mitochondrial and nuclear) and eventually carcinogenesis. Oxidative stress is due to the imbalance between production of free radicals and defense system of the body. Defense system of the body could function via enzymatic pathways such as superoxide dismutase, catalase, etc. or through non-enzymatic pathways using exogenous antioxidants like vitamins (A, C and E), flavonoids and glutathione (as thiol containing antioxidant). Foods containing natural antioxidants as useful regimens are capable of protecting the cells from the probable damages associated with free radicals. The incidence of above mentioned disorders is closely related to the intake of dietary antioxidants. However, there is a strong demand for discovery and development of potent antioxidants (3, 4 ). Chalcones or 1,3-diphenyl-2-propen-1-ones as precursor compounds in flavonoid biosynthesis in plants are one of the major categories of natural products with widespread distribution in spices, tea, beer, fruits and vegetables. Recently, natural chalcones and synthetic derivatives are subject of great interest for their pharmacological activities. Chemically they consist of open-chain flavonoids in which the two aromatic rings are joined by a three-carbon α,β-unsaturated carbonyl system (Figure 1) (5). Natural chalcones and also its synthetic derivatives have attracted increasing attention due to various pharmacological applications. They have exerted a broad spectrum of pharmacological properties such as antimalarial, anticancer, antimitotic, antiparasitic, anti-inflammatory, antibacterial, antifungal, anticonvulsant and antioxidant effects. They have also shown inhibitory activity towards some enzymes, especially mammalian alpha-amylase, cyclo-oxygenase (COX) and monoamine oxidase (MAO) (Figures 2, 3 and 4) (6-15). 

According to the recent reports about the efficacy of pyridine derivatives and also antioxidant effects of chalcones, we encouraged to design a new seires of chalcones containing pyridine moiety (16-18). In the other words, introduction of a pyridine ring was carried out in the structure of chalcone towards achieving novel and potent antioxidant agents.

## 2. Objectives

In the present study, a new series of chalcone with focusing on pyridine derivatives was synthesized. Antioxidant activity of synthesized derivatives were investigated using four antioxidant procedures consisting DPPH free radical scavenging, ferrous ion chelating (FIC) activity, beta carotene bleaching (BCB) and also Trolox equivalent antioxidant capacity (TEAC).

## 3. Materials and Methods

### 3.1. Reagents and Materials

All chemical substances consisting starting materials, reagents and solvents were purchased from the commercial supplier like Merck and Sigma-Aldrich companies. The purity of the prepared compounds was proved by thin layer chromatography (TLC) using various solvents of different polarities. Merck silica gel 60 F_254_ plates were used for analytical TLC. Column chromatography was applied on Merck silica gel (70-230 mesh) for purification of synthesized compounds. ^1^H-NMR spectra were recorded using a Bruker 400 MHz spectrometer and chemical shifts are expressed as δ (ppm) with tetramethylsilane (TMS) as internal standard. Deutrated solvents such as chloroform (CDCl_3_) or dimethylsulfoxide (DMSO-d_6_) were used for acquisition of NMR spectra. The IR spectra were obtained on a Shimadzu 470 spectrophotometer using potassium bromide (KBr) disks. Melting points were determined using Electrothermal 9001 elemental analyzer apparatus and are uncorrected. The mass spectra were run on a Finigan TSQ-70 spectrometer (Finigan, USA) at 70 eV. β-Carotene and 1,1-diphenyl-2-picryl-hydrazyl (DPPH) were purchased from Sigma-Aldrich. Linoleic acid, ferrous chloride, dimethyl sulfoxide (DMSO), chloroform, Tween ® 40, butylated hydroxytoluene (BHT) and activated MnO_2_ were purchased from Merck, ascorbic acid from VWR, ferrozine iron reagent, Trolox (6-hydroxy-2,5,7,8-tetramethylchroman-2-carboxylic acid) from Acros Organics and ABTS (2,2’-azinobis (3-ethylbenzothiazoline-6-sulfonic acid) diammonium) from Fluka.

### 3.2. General Procedure for Synthesis of Compounds 3a-3j

**Figure 5.**
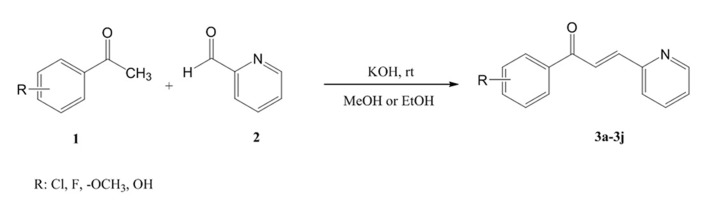
Synthetic Pathway of Compounds 3a-3j

According to Figure 5, in a flat bottomed flask, equimolar quantities of pyridine-2-carbaldehyde and various derivatives of acetophenone were mixed in methanol solvent and then one KOH pellet was added to the reaction medium. The reaction mixture was stirred at room temperature for 24 hours (14). After confirmation of the reaction end by thin layer chromatography (TLC), cold water (40 mL) was added and the formed precipitate was filtered and washed by diethyl ether (Et_2_O) and n-hexane. Column chromatography was applied for purification (EtAc/Petroleum ether, 60/40) of obtained final products. Spectral data of synthesized compounds 3a-3j are provided as following: 

(E)-1-(2-Chlorophenyl)-3-(pyridin-2-yl) prop-2-en-1-one (3a):

Yellow powder, mp: 192-194˚C, Yield: 66%, IR (KBr, cm^-1^) ῡ: 3039, 2922, 2880, 1710, 1580, 1460, 1082, 798.

(E)-1-(3-Chlorophenyl)-3-(pyridin-2-yl)prop-2-en-1-one (3b):

Creamy powder, mp: 190-193˚C, Yield: 61%, IR (KBr, cm^-1^) ῡ: 3400, 2860, 1710, 1670, 1620, 1080, 830.

(E)-1-(4-Chlorophenyl)-3-(pyridin-2-yl)prop-2-en-1-one (3c):

Yellow powder, mp: 124˚C, Yield: 38 %, IR (KBr, cm^-1^) ῡ: 3431, 3057, 2922, 1675, 1589, 1570, 1489, 1473, 1436, 1400, 1219, 1091, 1012, 1129, 760.

(E)-1-(2-Methoxyphenyl)-3-(pyridin-2-yl)prop-2-en-1-one (3d):

Yellow powder, mp: 66˚C, Yield: 54%, ^1^H NMR (400 MHz, CDCl_3_) δ: 3.92 (s, 3H, -OCH_3_), 7.01 (d, 1H, *J* = 8 Hz, H_3_-4-Methoxyphenyl), 7.54 (t, 1H, *J* = 8 Hz, H_5_-4-Methoxyphenyl), 7.28 (m, 1H, H_5_-Pyridine), 7.5 (m, 2H, H_3,4_-Pyridine), 7.63 (d, 1H, *J* = 16 Hz, -CH=CHCO-), 7.66 (d, 1H, *J* = 8 Hz, H_6_-4-Methoxyphenyl), 7.74 (t, *J* = 8 Hz, H_4_-4-Methoxyphenyl), 7.84 (d, 1H, *J* = 16 Hz, -CH=CHCO-), 8.68 (d, 1H, H_6_-Pyridine). IR (KBr, cm^-1^) ῡ: 3070, 3007, 2940, 2841, 1654, 1616, 1595, 1485, 1467, 1436, 1335, 1249, 1106, 1028, 1015, 927, 760.

(E)-1-(3-Methoxyphenyl)-3-(pyridin-2-yl)prop-2-en-1-one (3e):

Orange powder, mp: 143˚C, Yield: 55%, IR (KBr, cm^-1^) ῡ: 3392, 3045, 1690, 1600, 1085, 1045, 839, 742, 705. MS (*m/z*, %): 239 (M^+^, 5), 210 (10), 135 (15), 108 (30), 79 (15), 59 (100).

(E)-1-(4-Methoxyphenyl)-3-(pyridin-2-yl)prop-2-en-1-one (3f):

Yellow powder, mp: 69˚C, Yield: 68%, ^1^H NMR (400 MHz, CDCl_3_) δ: 3.89 (s, 3H, -OCH_3_), 6.98 (d, *J* = 8 Hz, H_3,5_-4-Methoxyphenyl), 7.29 (t, *J* = 4 Hz, 1H, H_5_-Pyridine), 7.47 (d, *J* = 8 Hz, -CH=CHCO-), 7.72 (d, *J* = 8 Hz, H_3_-Pyridine), 7.77 (d, *J* = 8 Hz, -CH=CHCO-), 8.12 (d, *J* = 8 Hz, H_2,6_-4-Methoxyphenyl), 8.15 (d, *J* = 8 HZ, H_4_-Pyridine) 8.68 (d, *J* = 4 Hz, 1H, H_6_-Pyridine). IR (KBr, cm^-1^) ῡ: 3041, 3068, 2933, 1660, 1597, 1510, 1427, 1334, 1263, 1170, 1018, 815, 777, 582. 

(E)-1-(2-Hydroxyphenyl)-3-(pyridin-2-yl)prop-2-en-1-one (3g)

Orange powder, mp: 108˚C, Yield: 39%, IR (KBr, cm^-1^) ῡ: 3057, 2924, 1710, 1680, 1587, 1475, 1450, 1149, 1082, 1055, 754.

(E)-1-(3-Hydroxyphenyl)-3-(pyridin-2-yl)prop-2-en-1-one (3h):

Yellow powder, mp: 93˚C, Yield: 37%, ^1^H NMR (400 MHz, DMSO-d_6_) δ: 7.08 (d, 1H, *J* = 8 Hz, H_4_-3hydroxyphenyl), 7.40 (t, 1H, *J* = 8 Hz, H_5_-3-Hydroxyphenyl), 7.44-7.48 (m, 1H, H_3_-Pyridine), 7.45 (s, 1H, H_2_-3hydroxyphenyl), 7.57 (d, 1H, *J* = 8 Hz, H_6_-3hydroxyphenyl), 7.71 (d, *J *= 12 Hz, -CH=CHCO-), 7.91 (m, 2H, H_4,5_-Pyridine), 8.69 (d, 1H, H_6_-Pyridine), 8.09 (d, *J *= 12 Hz, -CH=CHCO-), 10.01 (brs, OH). IR (KBr, cm^-1^) ῡ: 3055, 2960, 1664, 1616, 1591, 1581, 1475, 1452, 1345, 1288, 1211, 1193, 1093, 981, 771. MS (m/z, %): 225 (M^+^, 75), 196 (100), 180 (30), 132 (40), 104 (28), 78 (25). 

(E)-1-(2-Fluorophenyl)-3-(pyridin-2-yl)prop-2-en-1-one (3i):

Orange powder, mp: 145-148˚C, Yield: 63%, IR (KBr, cm^-1^) ῡ: 2922, 2856, 1685, 1560, 1458, 1085, 798.

(E)-1-(4-Fluorophenyl)-3-(pyridin-2-yl)prop-2-en-1-one (3j):

Yellow powder, mp: 100˚C, Yield: 68%, IR (KBr, cm^-1^) ῡ: 3429, 3080, 2880, 1676, 1595, 1506, 1460, 1226, 1157, 1085, 830, 540. MS (m/z, %): 227 (M^+^, 25), 198 (60), 123 (100), 106 (25), 95 (60), 78 (30), 51 (20).

### 3.3. Antioxidant Activity

#### 3.3.1. DPPH Radical Scavenging Activity

The radical scavenging activity was measured using the method of Hatano et al. with some modifications (19). Summarily, 0.2 mM solution of DPPH in methanol was prepared and 1.5 mL of this solution was added to the equal volume of each of test samples dissolved in methanol at different concentrations. After shaking, the mixture was maintained in dark for 30 min. Then, the absorbance was measured at 517 nm against a blank. Ascorbic acid and butylated hydroxyanisole (BHA) were used as standard references. The scavenging activity was calculated using the formula:

Scavenging Activity (%) = [(A_517_ of control - A_517_ of sample) / A_517_ of control] × 100.

#### 3.3.2. Ferrous ion Chelating Activity

Ferrous iron-ferrozine complex method with some modification was used for determination of chelating activity of samples for ferrous ions Fe^2+ ^(20). Briefly, 25 µL of FeCl_2 _solution (2 mM) was added to a mixture containing 1.5 mL of H_2_O and 2 mL of the test samples in methanol at different concentrations. The reaction was proceeded by adding 50 µL ferrozine solution (5 mM) after 30 seconds. The mixture was shaken well and incubated for 10 minutes at room temperature. Absorbance of the solution was then measured at 562 nm. Quercetin was used as positive control. The ability of the extracts and fractions to chelate ferrous ion was calculated using the equation described above for DPPH.

#### 3.3.3. Inhibition of β-carotene Bleaching

Antioxidant activity of the samples was determined according to a slightly modified version of the β-carotene bleaching method (21). In this study 5 mg of β-carotene was dissolved in 10 mL of chloroform. 33 µL of linoleic acid, 750 µL of β-carotene solution and 225 mg of Tween 40 were mixed. The solvent was completely removed using a rotary evaporator. Then 75 mL of oxygenated distilled water was added and the mixture was emulsified for 15 min in a sonicator to form an emulsion A. Aliquots of 3.5 mL of this emulsion were transferred into a series of stopper test tubes containing 1 mL of samples dissolved in ethanol in various concentrations. Optical density (OD) at 470 nm was determined for all samples immediately (t = 0) and at the end of the time period (t = 120). A second emulsion was also prepared and used as blank to zero the spectrophotometer. This emulsion consisted of 50 mL of oxygenated water, 22 µL of linoleic acid and 150 mg of Tween 40. The percentage inhibition was calculated according to the following formula:

Inhibition (%) = [(A_A(120)_ – A_C(120)_) / (A_C(0)_ – A_C(120)_)] × 10

In which A_A(120) _is the absorbance of the sample at t = 120 min, A_C(120)_ is the absorbance of the control at t = 120 min and A_C(0) _is the absorbance of the control at t = 0 min.

#### 3.3.4. Trolox Equivalent Antioxidant Capacity (TEAC)

Antioxidant capacity of the samples (compounds 3a-3j) was measured with some modifications (22). In this procedure ABTS (54.2 mg) was dissolved in phosphate buffer (pH = 7) and activated to ABTS^+^ radical by addition of 100 mg MnO_2_ with stirring for 30 minutes. Then, the solution was centrifuged (5 min, 10000 rpm), filtered (0.22 µm) and diluted with phosphate buffer so that A_0_ = 0.7. Then, 2 mL of ABTS^+^ radical was added to test tubes containing 1 mL of samples dissolved in ethanol in various concentrations. Time of reaction was 20 minutes. Absorbance of the solution was measured at a wavelength of 734 nm and antioxidant capacity of the samples was calculated according to the following formula:

Inhibition, % = [(A0 - A1) / A0] × 100

Where A_0_: the absorbance of the control, A_1 _: the absorbance of the sample. Trolox was used as standard.

### 3.4. Statistical Analysis

The experimental results were performed in triplicate. The data was recorded as Mean ± standard deviation and analyzed by SPSS (version 16 for Windows Xp.). Non-parametric Friedman test was performed by the following procedures and P < 0.05 was regarded as significant.

## 4. Results

### 4.1. Synthesis

According to the scheme 1, all compounds 3a-3j were synthesized through an aldol condensation procedure in the presence of potassium hydroxide (KOH) in methanol (MeOH) or ethanol (EtOH) solvent. The intended reactions run at room temperature for 24 hours. The related product was obtained via an aqueous work-up with an average yield (37-68%). A yellowish to orange powder were obtained for each compound. ^1^H NMR, IR and MS spectroscopic procedures were applied for characterization and confirmation of synthesized derivatives. According to the NMR data, a coupling constant equal to 12 Hz (J_*trans*_) was obtained for vinylic hydrogens of the double bond. This coupling constant confirmed the *E* isomer of the synthesized derivatives. Potassium bromide disk was prepared for each sample before IR spectroscopy. The related melting points were also measured and a range of 66-194˚C was obtained in this series. Methoxy derivatives showed the lowest melting point and chlorinated derivatives rendered the highest melting point.

### 4.2. Antioxidant Activity

All compounds 3a-3j were synthesized and evaluated for antioxidant activity via four different antioxidant procedures (Table 1). None of them rendered suitable and significant antioxidant effects in BCB as well as DPPH free radical scavenging methods. Totally, synthesized compounds 3a-3j showed superior antioxidant property compared to quercetin in FIC test. Compound 3e with meta substitution of methoxy group exerted the highest antioxidant capacity (16.53 ± 1.21 µg/mL) in these series in FIC test. Compounds 3g (58.85 ± 1.10 µg/mL) and 3i (58.73 ± 12.94 µg/mL) with ortho substitution of hydroxyl and fluorine moieties respectively, were also demonstrated higher antioxidant activity in comparison with quercetin (87.24 ± 3.93 µg/mL). Compounds 3g (4.82 ± 0.11 µg/mL) and 3h (6.33 ± 0.30 µg/mL) were also active antioxidant agents in TEAC method but with lower potency than Trolox as reference agent.

**Table 1.** Results (EC_50_ ± SD, µg/mL) of Antioxidant Assay of Compounds 3a-3j

Compounds	R	DPPH Free Radical Scavenging	BCB	Fe^2+ ^Chelating Activity	TEAC
**3-a**	2-Cl	> 250	146.56 ± 4.97	> 250	47.75 ± 0.52
**3-b**	3-Cl	> 250	> 250	> 250	> 250
**3-c**	4-Cl	> 250	> 250	> 250	> 250
**3-d**	2-OCH_3_	> 250	81.97 ± 21.58	> 250	> 250
**3-e**	3-OCH_3_	> 250	> 250	16.53 ± 1.21	> 250
**3-f**	4-OCH_3_	> 250	> 250	> 250	> 250
**3-g**	2-OH	155.70 ± 3.44	23.21 ± 1.17	58.85 ± 1.10	4.82 ± 0.11
**3-h**	3-OH	> 250	> 250	> 250	6.33 ± 0.30
**3-i**	2-F	> 250	82.81 ± 8.20	58.73 ± 12.94	27.75 ± 0.36
**3-j**	4-F	> 250	42.53 ± 4.01	> 250	30.71 ± 0.26
**Vit C**	-	16.07 ± 0.28	-	-	-
**BHT ** ^**a**^	-	18.81 ± 0.57	1.65 ± 0.94	-	-
**Quercetin**	-	-	-	87.24 ± 3.93	-
**Trolox**	-	-	-	-	3.83 ± 0.22

^a^ Abbreviations: BHT, butylatedhydroxytoluene.

### 4.3. Statistical Analysis

The results of Friedman test showed one of the assays (DPPH) has significantly different EC_50_ values in comparison with the others and there is no significant difference among the results of three other methods (BCB, TEAC & FIC).

## 5. Discussion

A new series of pyridine based chalcone was synthesized and their antioxidant properties were assessed by four antioxidant procedures. All compounds 3a-3j were synthesized with an acceptable and average yield at room temperature via an aldol condensation. Fortunately, compounds 3e (16.53 ± 1.21 µg/mL), 3g (58.85 ± 1.10 µg/mL) and 3i (58.73 ± 12.94 µg/mL) showed higher antioxidant activity (EC_50_ ± SD) than quercetin (87.24 ± 3.93 µg/mL) as reference antioxidant agent in ferrous ion chelating (FIC) method. Compounds 3g (4.82 ± 0.11 µg/mL) and 3h (6.33 ± 0.30 µg/mL) also exhibited an acceptable antioxidant property compared to Trolox (3.83 ± 0.22 µg/mL) in TEAC method. None of the synthesized compounds demonstrated significant antioxidant activity in DPPH free radical scavenging as well as beta carotene bleaching tests. Compounds 3e, 3g as well as 3i could be suggested as potential antioxidant lead compounds.
